# How Big Data and High-performance Computing Drive Brain Science

**DOI:** 10.1016/j.gpb.2019.09.003

**Published:** 2019-12-02

**Authors:** Shanyu Chen, Zhipeng He, Xinyin Han, Xiaoyu He, Ruilin Li, Haidong Zhu, Dan Zhao, Chuangchuang Dai, Yu Zhang, Zhonghua Lu, Xuebin Chi, Beifang Niu

**Affiliations:** 1 Computer Network Information Center, Chinese Academy of Sciences, Beijing 100190, China; 2 University of Chinese Academy of Sciences, Beijing 100190, China; 3 Guizhou University School of Medicine, Guiyang 550025, China; 4 Center of Scientific Computing Applications & Research, Chinese Academy of Sciences, Beijing 100190, China

**Keywords:** Brain science, Big data, High-performance computing, Brain connectomes, Deep learning

## Abstract

**Brain science** accelerates the study of intelligence and behavior, contributes fundamental insights into human cognition, and offers prospective treatments for brain disease. Faced with the challenges posed by imaging technologies and **deep learning** computational models, **big data** and **high-performance computing** (HPC) play essential roles in studying brain function, brain diseases, and large-scale brain models or connectomes. We review the driving forces behind big data and HPC methods applied to brain science, including deep learning, powerful data analysis capabilities, and computational performance solutions, each of which can be used to improve diagnostic accuracy and research output. This work reinforces predictions that big data and HPC will continue to improve brain science by making ultrahigh-performance analysis possible, by improving data standardization and sharing, and by providing new neuromorphic insights.

## Introduction

A human brain has about 100 billion neurons [1]. Each neuron must pass through approximately 10^15^ neurons to communicate with another neuron [2]. The brain is responsible for human intelligence. Compromised functioning of the brain resulting from brain disease causes more than 6.3% of the global disease burden in terms of disability-adjusted life years. Moreover, the World Health Organization (WHO) noted that the proportion of brain disease in the global disease burden is projected to increase by 12% from 2005 to 2030 [3]. In 2010, around $2.5 trillion spent on research on brain disease, an amount that is estimated to increase to $6 trillion by 2030 [4]. According to statistics from the WHO, brain diseases account for 12% of the world's total deaths [5]. Also, in low- and middle-income countries and high-income countries, encephalopathy accounts for 16.8% and 13.2% of the total death toll, respectively [5].

Driven by the rising incidence of brain diseases, brain research is important for understanding brain function mechanisms, promoting the diagnosis and treatment of brain diseases, and improving the development of brain-like intelligence. In 2013 [6], the European Union announced the Human Brain Project (HBP) to strengthen neuroscience research and gain a comprehensive understanding of brain function through worldwide research [6]. Moreover, the USA, China, and many other countries and organizations had also focused on and invested in brain-research projects (Table 1). The governments of these countries had attempted to build platforms for studying the human brain using neuroinformatics, brain simulation, and brain-tissue science to create a validation model that runs on supercomputers [7].

**Table 1:** **National brain projects**




*Note*: Brain/MINDS, Brain Mapping by Integrated Neuro technologies for Disease Studies; MIRI, Multi-Investigator Research Initiative; PSG, platform support grants.

**Figure 1:**
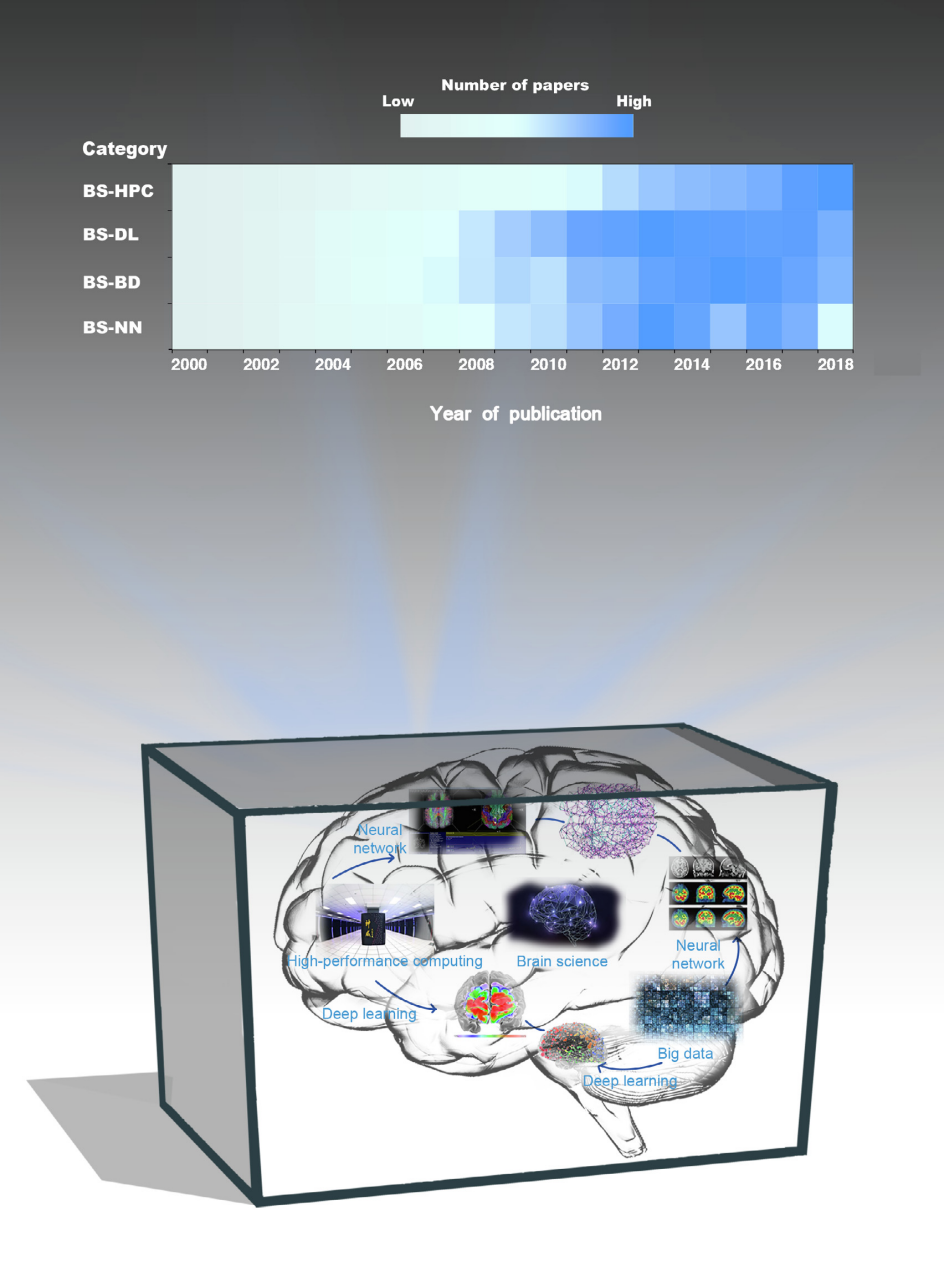
**Research overview of big data and HPC methods in brain science** **A**. The heatmap shows the changes in the number of articles published annually from 2000 to 2018 in four research directions of brain science: brain science with HPC; brain science with deep learning; brain science with big data; and brain science with neural networks. Articles in brain science with deep learning, brain science with big data, and brain science with neural networks reached their highest numbers in 2013, whereas articles in brain science with HPC reached its highest number in 2018. All articles were retrieved by searching using keywords “brain science, HPC” (BS-HPC), “brain science, deep learning” (BS-DL), “brain science, big data” (BS-BD), or “brain science, neural network” (BS-NN) in Google Scholar in September 2019. **B**. Combinations between brain science and big data or HPC methods. Big data provide a wealth of knowledge and data, from which neural networks and deep learning methods can extract features that represent brain functions, mechanisms, or diseases. Big data can also be used to build computational models. HPC provides storage space and formidable computing power for the study of brain science. HPC, high-performance computing.

The development of big data technology and HPC has contributed to insights gained from these brain-research projects. Big data methods have improved the details of brain scans in several ways, thereby laying the foundation for the generation of new knowledge that can drive understanding of the human brain even further. HPC methods have advanced data storage, computational accuracy, and computational speed, and thereby assist in the processing of vast and very complex data sets. The development and popularization of brain science, big data, and HPC methods are shown in Figure 1. Data, methods, and computing power are being continuously added to brain science research. Particularly after 2006, brain science combined with big data and deep learning has become a research hotspot. Subsequently, the support of neural networks and HPC for brain science research has also been enhanced considerably. An overall increase in interdependence has been observed from 2000 to 2018.

## Combination and revolution

**Figure 2:**
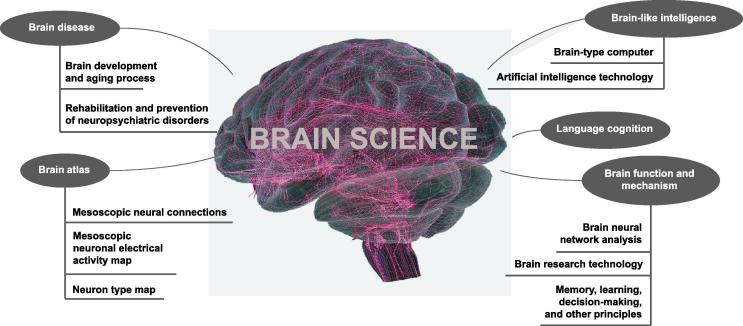
**General classification of research activities in brain science** This figure shows the main research directions in contemporary brain science.

Brain science is based primarily on biological insights and data-driven, bottom-up experimental research. Figure 2 shows the current research fields of brain science: brain function/mechanisms, diagnosis of brain disease, brain-like intelligence, and refinement of sub-areas. These seemingly different research areas are interrelated. In biology, “classical reductionism” suggests that each entity comprises smaller parts. That is, the aging process, decision-making principles, pathogenesis, or brain cognition are the “macroscopic” consequences of “microscopic” behavior and reaction in the brain. In this sense, “microscopic” denotes less vision and more knowledge and, most importantly, big data. In addition, these connections between the microscopic parts are usually linear [8], and there are approximately 10^15^ such linear connections in the brain [9]. Thus, exploring microscopic characteristics in such a large number of structures and complex connections results in extensive use of computational resources. Moreover, as brain science advances, the resolution becomes infinite.

The cycle of acquisition of functional magnetic resonance imaging (fMRI) data has accelerated from 4 s to 1 s in speed, and the resolution has increased from 5 mm^3^ to 1 mm^3^ [10]. Faster speeds and more detailed visions bring challenges while bringing amazing scientific discoveries. For example, genome-wide association studies combined with functional and structural brain-imaging phenotypes to verify genes for iron transport and storage are associated with the magnetic susceptibility of subcortical brain tissue [11]. However, a single genome contains around 180 GB of uncompressed data that is equivalent to 30 copies of 3 billion bases, and 100 GB of compressed information needs to be retained over time [12]. Therefore, the ability to process data analysis and workflows, meet data storage/simulation requirements, and even break the limits of computational speed are critical factors in brain science. Here, big data and HPC bear the brunt of these challenges. Advances in data storage and mining technologies allow users to retain increasing amounts of data directly or indirectly and to analyze such data for valuable discoveries [13]. HPC provides a high-speed computing environment that meets high-throughput and multitasking computing features, including the use of clusters of multiple processors or computers as part of a single machine [14].

**Figure 3:**
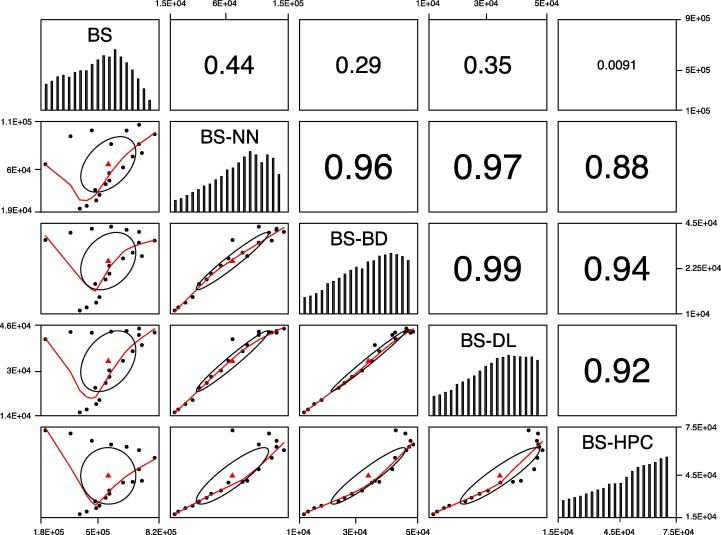
**Research status of brain science in combination with other fields** This figure shows the number of articles listed in Google™ Scholar each year from 2000 to 2018 by the following terms: BS, BS-NN, BSBD, BS-DL, and BS-HPC. This figure comprises three parts. The histogram shows trends in the number of articles on BS combined with each of the other four fields. Each thumbnail in the lower triangular area consists of a correlation ellipse, a scattergram of the corresponding rows and columns, and its LOWESS smoothing curve. The correlation ellipse indicates the correlation between corresponding rows and columns. A flatter oval indicates a stronger correlation. The LOWESS smoothing curve shows the trend between the two sets of data over time. Each thumbnail in the upper triangle contains a value that represents the correlation coefficient of the corresponding row and column. For example, the value 0.44 in the first row and the second column refers to the correlation coefficient between BS and BS-NN. BS, brain science; BS-NN, brain science with neural network; BS-BD, brain science with big data; BS-DL brain science with deep learning; BS-HPC, brain science with high-performance computing.

In recent decades, research funding agencies and scientists have placed great importance on the use of big data and HPC techniques in brain science. With regard to big data, the HBP’s fifth sub-project is to build a Neuroinformatics Computing Platform (NCP) for integrating multidimensional brain data (*e.g.*, molecules, genes, cells, networks) and provide powerful analysis of brain data to simplify models of actual neuronal circuits [15]. The Allen Human Brain Atlas is a unique multi-modal atlas that maps gene expression across adult brains [16], including magnetic resonance imaging (MRI), diffusion tensor imaging, histology, and gene-expression data [17]. The BrainSpan Atlas of the developing human brain is a unique resource for exploring the development of the human brain. It comprises in situ hybridization data, RNA sequencing, microarray data, as well as broad and detailed anatomical analysis of gene expression during human brain development [17]. The seventh sub-project of the HBP has been to establish a platform for high-performance analytics and computing designed to provide the HBP and the neuroscience community with HPC systems to meet their particular [18]. Currently, the HBP has four tier-0 supercomputer centers: Barcelona Supercomputing Centre, Cineca, Centro Svizzero di Calcolo Scientifico and Jülich Supercomputing Centre (JSC) [6]. The supercomputer JUROPA at the JSC has developed an ultrahigh-resolution three-dimensional (3D) human brain model called BigBrain [19]. An account of research in brain science combined with other fields is shown in Figure 3. The amount of literature on brain science that encompasses the other four fields detailed in Figure 3 increased from 2000 to 2018. The correlation coefficients in Figure 3 demonstrate that these combinations have stimulated productivity in brain science.

## Research advances enabled by big data and HPC

### Function and mechanisms within the brain

Brain activity consists of dynamic sets of input sensations and spontaneous responses [20]. However, the brain is very active even in the absence of explicit inputs or outputs [21]. Researchers often create brain-simulation models (*e.g.*, simulations of brain molecules and brain cells) or use fMRI data of living human brains to gain insight into brain structure. For example, one MRI time-course of 512 echo-planar images in a resting human brain obtained every 250 milliseconds showed fluctuations of physiologic origin in signal intensity in each pixel. Such data can reveal functional connections in the motor cortex of the resting human brain [22]. Furthermore, recent functional imaging studies have revealed co-activation in a distributed network of cortical regions that characterize the quiescent state or “default mode” of the human brain [23]. Subtle imaging provides a large data set that requires a large storage space and capability to undertake high-resolution analysis [24]. Applications that mimic brain structures could simulate about 100 billion neurons at the molecular level, and each requires 0.1 megabyte (MB) to 10 terabytes (TB) of memory [25]. The fMRI divides the brain into tens of thousands of voxels and then images the entire brain continuously with high time resolution. One scan is used as a processing unit, so the entire brain generates a very large amount of high-dimensional data [26]. Although extant computational models often carry out feature combinations (*e.g.*, Suk et al. [27]) in studies on brain function and brain mechanisms and even multimodal fusions of features (Liu et al. [28]) to reduce “dimensional disasters”, their capacity to leverage computational power to analyze highly multidimensional data is limited.

Deep learning computational models use eigenvector sets to represent biological information. This strategy enables computational models that consist of multiple processing layers to learn data representations with multiple levels of abstraction [29]. Li et al. [30] used image-analysis technology combined with a Statistical Parametric Mapping (SPM) and Voxel-Based Morphometry (VBM) toolbox to visualize cerebrospinal fluid and other brain components. Besides, Vidaurre et al. [31] proposed a framework that used a hidden Markov model to infer a consistent and interpretable dynamic brain network in different data sets. Spitzer et al. [32] proposed a model based on a convolution neural network (CNN) to predict 13 regions of the human visual system. Even though deep learning provides important insights, the deep neural network is a complex hierarchical structure similar to a biological neural network. Each layer consists of several “artificial neurons”; the more layers in the neural network, the better the insights. However, mapping relationships from input layers to output layers often reveals non-linear relationships, and some deep learning models may even extend to brain mechanisms at the 3D level (*e.g.*, Payan et al. [33], Hosseini-Asl [34]).

Whether using data or computational models, it is clear that the exploration of brain function and brain mechanisms demands a great deal of storage capacity and powerful computing capabilities. Big data and HPC approaches are, therefore, necessary in all brain science studies that generate such data. This is especially true if supercomputers are used to model brain function. For instance, Apache Spark with autoencoder for fMRI [35], and JUROPA in JSC have both implemented ultrahigh-resolution 3D models of the human brain [36], thereby greatly improving computational performance and the speed of data processing. In addition, researchers in China have used the Tianhe-1A supercomputer for NEST (NEural Simulation Tool), which showed that a single compute node implements a neural-network simulation of 7.3 × 10^4^ neurons and implementation of 5.6 × 10^6^ neurons on 128 compute nodes [12]. Use of an NVIDIA graphic processing unit (GPU) accelerates the Groningen MAchine for Chemical Simulation (GROMACS) [37] by a factor of 3–4, thereby reducing the time spent on simulations of molecular dynamics in the brain from days to hours. HPC helps virtual epilepsy (VEP) brain model to explore system parameter space, fit and validate brain model, thus promoting large-scale brain models to encourage the development of personalized treatment and intervention strategies [38]. HPC methods not only allow new studies on brain science to overcome traditional hardware and software constraints, they also foster a new research dynamic whereby studies of intrinsically biological problems can be studied by relying on HPC methods only. The advantage of this development is that HPC methods enable in-depth quantification that was impossible previously [19]. For instance, the Salk Institute has established a 3D molecular computational model to understand the human brain through neural communication processes in the ciliary ganglion of the chicken, which model adjusts data parameters with the aid of a central HPC system in the San Diego Supercomputer Center.

### Research on brain disease

The diagnosis and treatment of brain diseases—especially Alzheimer’s disease (AD) and Parkinson’s disease (PD)—is an important focus of clinical research into brain disease in many countries. AD is a progressive neurologic disease that presents as memory and cognitive dysfunctions [39]. Major clinical manifestations include memory impairment, aphasia, agnosia, altered personality, and behavior changes [40]. The failure rate of treatment in the human brain in clinical trials for AD reached 99.6% from 2002 to 2013 [41]. In 2010, the total number of people with dementia globally was approximately 35.6 million. This figure is expected to double every 20 years to reach 65.7 million in 2030 and 115.4 million in 2050 [42]. PD is a common dyskinetic disease in the elderly that affects 1.5–26 per 100,000 of the general adult population [43]. Projections suggest that the number of patients with PD aged over 50 years may reach 8.7–9.3 million worldwide by 2030 [44]. Research on brain disease has become a high priority in several countries. The Japan Brain Project [45] seeks to improve the understanding of human brain diseases such as AD and schizophrenia via experimentation on marmoset brains. The China Brain Project seeks to study pathogenic mechanisms and to develop efficacious diagnostic and therapeutic approaches for developmental disorders of the brain (*e.g.*, autism, intellectual disabilities), neuropsychiatric disorders (*e.g.*, depression, addiction), and neurodegenerative disorders (*e.g.*, AD, PD) [46]. The urgency to reduce the escalating societal burden associated with these disorders and the ineffectiveness of current therapies has resulted in calls for early diagnoses at pre-symptomatic and prodromal stages so that early intervention may halt or delay disease progression [46].

With a more profound understanding of brain function and mechanics driven by big data and HPC approaches, researchers are better able to diagnose and treat disease. More information about brain diseases is key to improving the diagnosis and treatment of brain diseases, so it is necessary to use big data and HPC methods to build detailed and effective disease computational models. For example, big data collected from traditional medical imaging or advanced wearable sensing devices (see Shamir et al. [47]) has produced huge volumes of information. Also, Gupta and colleagues [48] used an autoencoder to understand the features of 2D patches through MRI. Yang and co-workers [49] used MRI data from the Open Access Series of Imaging Studies and Alzheimer’s Disease Neuroimaging Initiative (ADNI) to develop a classification of AD. Machine learning and deep learning methods for disease diagnosis (*e.g.*, support vector machine (SVM) [50], Gaussian kernel SVM [51], enhanced logistic regression model [52], deep belief networks [53]) have also been found to be indispensable in improving efficiency. Big data combined with deep learning models not only provide increased information generation and analytical efficiency but also improve accuracy. Experimental accuracy with respect to AD, mild cognitive impairment (MCI), and MCI-converter diagnoses have reached 95.90% in the ADNI data set [25]. Moreover, Sarraf et al. [54] used fMRI data with CNN and LeNet-5 to diagnose AD, and achieved an accuracy of 96.86% using test data. Payan [28] used 3D-CNN to achieve accuracy ≤95.39% when classifying patients as AD or healthy controls.

While big data and deep learning methods have yielded notable benefits, they have also introduced new computational challenges that can be resolved only by HPC methods. The machine from Hewlett–Packard Development Company contains 160 TB of single memory (equivalent to 160 million books of active memory). This helps the German Center for Neurodegenerative Diseases speed up the genomics pipeline by nine times, meaning that the process would have taken only approximately 36 seconds instead of the original 25 min [22]. Moreover, a research team at the Friedrich-Alexander-Universität (Germany) used a supercomputer from the Regionales Rechenzentrum Erlangen to carry out all-atom molecular dynamics simulations in an explicit solvent of 0.7 μs in total on five Aβ9-42 oligomers (monomers through to pentamers) to reveal Aβ peptides, an important factor in AD [55]. All in all, these examples show the indispensable nature of HPC approaches.

### Brain models and connectomes

The human brain is a complex multi-scale structure in space and time, and produces fine molecular, cellular and neuronal phenomena [56], [57]. Neuroimaging can provide brain images with high temporal and spatial resolution, but dynamic information for the brain is lacking. Therefore, in brain science research, simulation tools, brain models, and connectomes have been developed gradually and built to provide simulation information of neurons, brain structures, and networks. Simulation tools focus on individual neurons and the corresponding models of ion channels [58]. For example, the GEneral NEtwork SImulation System (GENESIS) was designed to simulate neural networks using standard and flexible methods to obtain detailed and realistic models [59]. A model of the human brain is a “reference brain” that provides important biological details. It outlines the spatial framework and brain composition from a macroscopic perspective and helps researchers extract and analyze microscopic data from molecular processes to various behaviors for modeling and simulation. The brain connectome is the “Google™ Maps” for brain models. It provides precise human brain coordinates and helps researchers transform detailed neural connections with human brain cognition and behavior. Connectomes map elements to human brain networks dynamically [60], where circuit abnormalities presumably reflect a complex interplay between genes and the environment [61]. Large-scale models of the human brain and connectomes not only provide basic insights of brain structure [62] but also serve as biomarkers of brain diseases to help researchers explain diseases such as AD and PD [63], [64], [65], [66], [67] and even help researchers understand the sex-based differences in human behavior [68].

Vast human brain structures and high-resolution imaging technology determine the essence of brain models and connectomes to be a big data set. The brain model of the HBP consists of 100 neocortical columns [69]. Defense Advanced Research Projects Agency (DARPA)’s Synapse project 500 billion neurons [70], [71], [72]. Also, Izhikevich et al. published a detailed large-scale thalamocortical model that simulates one million multicompartmental spiking neurons [73]. On a microscopic scale, the number of neurons and synapses contained in brain connectomes is approximately 10^10^–10^11^ and 10^14^–10^15^ [74]. At the macroscopic scale, the cortical hypothalamic junction contains hundreds of brain regions and thousands of comprehensive pathways data sets [75]. Currently, the Human Connectome Project (HCP) already has 7-T diffusion magnetic resonance imaging (dMRI) and 3-T resting-state fMRI (R-fMRI) data [76], [77], [78]. Not only structure but also high-resolution imaging. When building a brain model, an optical microscope sufficient to track a single neuron has a resolution of 0.25–0.5 microns, and an electron microscope capable of displaying synaptic or chemical signals has a resolution of nanometers [79]. Diffusion tensor imaging [80] and four main MRI modes (structural MRI, task fMRI, dMRI, R-fMRI) can be used to measure connectivity in the brain [61] with resolution of 1–3 mm or even smaller [81]. Brain models and connectomes as big data sets provide abundant information and knowledge to drive the development of brain-research programs. The Izhikevich neuron model has influenced more than 3000 academic studies by 2019. Based on the 500-subject release of the HCP, the Budapest Reference Connectome Server v2.0 and v3.0 has generated the common sides of connectomes in 96 and 477 different cortical layers [82], [83]. Also, disease research by the HCP applies HCP-style data to people at risk or suffering from brain disease (*e.g.*, anxiety, depression, epilepsy) [84].

The human brain is not only a simple big data set, but also a complex mathematical object. Hence, building models of the human brain and connectomes requires powerful platforms for data storage and processing. To meet the HPC requirements for brain models, the Izhikevich neuron model [85] used the Beowolf Cluster [86] with 60 processors of 3-GHz each, the HBP and Synapse project used the IBM Blue Gene supercomputer, and Spaun used eight Core Xeon processors (2.53 GHz) [71]. The HCP infrastructure uses IBM HPCS from the WU Center for High Performance Computing to execute pipelines and user-submitted jobs to meet the high-throughput data-processing requirements of approximately 200,000 inputs and outputs per second [71], [87]. In addition, the HCP has established a set of informatics tools, including ConnectomeDB [88] and Connectome Workbench [89], to collect, process, share and visualize high-throughput data [90]. Not only brain models and connectomes, workflows and simulation tools are moving toward high-performance computing and distribution. Simulation tools such as GENESIS [59], NEURON [91], NEST [84], Network and Cache Simulator (NCS) [92], Neosim [93], and SpikeNET [94] have also been extended to support parallel processing systems to improve performance. After parallelization, NEURON achieved almost linear acceleration. It requires an integration time of 9.8 seconds and communication time of 1.3 seconds if running 40,000 realistic cells on 2000 processors on the IBM Blue Gene supercomputer [95]. Also, the BigBrain-based 3D-PLI workflow uses the JUDGE and JUROPA supercomputers from the JSC to meet the requirements for data reading, analysis, and calculation [96]. The JUROPA supercomputer owns 26,304 cores, and its Rpeak reaches 308.3 TP/s. With HICANN from Heidelberg University, brain activity can be simulated at 10,000 times the normal speed to compress one day into ten seconds [72]. The HBP estimates that supercomputers with exaflop computing speed and exabyte computing memory can simulate a complete human brain (1000 times that of the rodent brain) [69]. With the development of ultrahigh-performance computers and computing environments, a model of the whole brain and dynamic brain connectomes will be completed eventually.

## Prospects for brain science

Using big data and HPC methods to address problems in high-dimensional brain science research is important. Hence, research programs on brain science (and especially large government-led programs) are becoming more reliant on supercomputers and large databases. Table 1 introduces the basic concepts of some national brain programs, including commonly used platforms and supercomputers. Table 2 lists several large databases on brain science research. At this stage, governments and funding agencies have resolved economic problems, and several mature HPC environments (and some customized for brain science research) are available. However, three main limitations exist. First, computing resources are scattered. As shown in Table 3, Sequoia, K, JURECA, and Tianhe-1A are among the top-500 supercomputers in the world, and are ranked 13th, 20th, 52th, and 87th, respectively (June 2019). These supercomputers are in the USA, Japan, Germany, and China, respectively. In the near future, we hope that a cooperative high-performance platform, shared high-performance resources, and universal standards for analysis of brain science data can be created. The second limitation is based on storage capacity and computational performance. As shown in Figure 1, Figure 3, the correlation coefficient between brain science and the HPC is smaller than that of the others, but the research output in these fields has been increasing from 2000 to 2018. In contrast, although the correlation coefficient is larger, the number of published articles in brain science and other fields decreased slightly after 2013. These results could be because brain science using HPC is constantly evolving, but the support provided by HPC is insufficient and limits the further development of brain science with other fields. As one of the most important elements of the brain model, the amount of imaging data of a neuron can reach the petabyte (PB) level readily at a resolution of microns or even nanometers, which is far beyond that in the human genome. The Sequoia supercomputer can simulate 530 billion neurons and 137 trillion synapses, but this is less than 1% of the information-processing capacity of humans [10]. The K supercomputer analyzed approximately 1% of neuron connections in the simulated human brain; this implies that simulation of the entire brain neuron requires 100 times the power of the best supercomputer performance. The JSC has developed an algorithm that can simulate 100% of neuron connections but, unfortunately, no supercomputer can run this algorithm [97]. Alternatively, a neuromorphic computer that breaks the conventional Von Neumann architecture could be developed [98] or even a combination of traditional supercomputers with neuromorphic computing could be initiated. The third limitation is the lack of randomness and dynamics. To fully grasp the relationship between the human brain and consciousness, emotions, and even thinking, a dynamic and complete brain model is, ultimately, what is needed. The “perfect” brain should be able to simulate all dynamic neural activity (not just static images), support the ability to switch visual imaging at any resolution, and even provide brain coordinates at any scale. This perfection requires not only sufficient storage space and flexible and variable computing environments, but also meticulous process design and impeccable visualization tools. To further meet random and dynamic requirements, these tools should be developed to be distributed and parallel, and they should even be highly portable.

**Table 2:** **Databases related to brain science**




*Note*: AD, Alzheimer's disease; BIRN, Biomedical Informatics Research Network; BP, bipolar disorder; MRI, magnetic resonance imaging; fMRI, functional MRI; tfMRI, task-based fMRI; HCP, Human Connectome Project; BCP, Baby Connectome Project; ABCD, Adolescent Brain Cognitive Development; OASIS, Open Access Series of Imaging Studies.

**Table 3:** **Supercomputers related to brain science**

In general, the development of brain science is destined to be inseparable from the support of big data and HPC. Big data, HPC and brain science promote each other and will break all the technical bottlenecks. A detailed and accurate atlas of the brain, and a fully simulated dynamic computer model will be produced through the use of big data and HPC methods, which could be used as an adjunct to the mouse model for developing new drugs associated with brain disease.

## Conclusion

Big data and HPC have become indispensable for: (i) exploration of brain function; (ii) determining the mechanisms of brain disease; (iii) building a whole-brain model and dynamic connectomes. Big data provides large databases on knowledge of the brain, such as the Allen Human Atlas, and efficient frameworks for big data analysis, such as Apache Spark. HPC methods use platforms such as the JSC and supercomputers such as Sequoia, and can solve the challenges of computational performance caused by large data sets and complex models. HPC methods require more storage space but provide increasingly powerful simulation capabilities to reduce runtimes for complex simulations from days to hours. Big data combined with deep learning models can increase the diagnostic accuracy of AD to more than 90%. HPC has also transformed biology into a science of deep quantitative analysis, and has made breakthroughs in characterizing neural communications.

Brain science will continue to develop in a more comprehensive, precise, and detailed direction with the support of governments and the scientific community. Over time, big data analysis for brain science will be standardized, HPC will be shared and coordinated, and new, ultrahigh-performance forms of computer will become a universal reality. However, as big data methods generate ever-larger pools of data, ever-faster and more powerful computational methods will be required to analyze them. Therefore, the “one-way ratchet” of this relationship suggests that brain science will become more reliant on big data and HPC methods in the future.

## Competing interests

The authors have declared no competing interests.
